# Development of Scheelite Tailings-Based Ceramic Formulations with the Potential to Manufacture Porcelain Tiles, Semi-Stoneware and Stoneware

**DOI:** 10.3390/ma13225122

**Published:** 2020-11-13

**Authors:** Julliana Marques R. de Figueirêdo, Fabiana Pereira da Costa, Jucielle Veras Fernandes, Alisson Mendes Rodrigues, Gelmires de Araújo Neves, Romualdo Rodrigues Menezes, Lisiane Navarro de Lima Santana

**Affiliations:** 1 Programa de Pós-Graduação em Ciência e Engenharia de Materiais (PPG-CEMat), Universidade Federal de Campina Grande, Av. Aprígio Veloso-882, Bodocongó, Campina Grande-PB 58429-900, Brazil; jullymrc@gmail.com (J.M.R.d.F.); fabiana_p.costa@hotmail.com (F.P.d.C.); jucielle_fernandes@hotmail.com (J.V.F.); gelmires.neves@ufcg.edu.br (G.d.A.N.); romualdo.menezes@ufcg.edu.br (R.R.M.); lisiane.navarro@ufcg.edu.br (L.N.d.L.S.); 2 Unidade Acadêmica de Engenharia de Materiais, Centro de Ciências e Tecnologia, Universidade Federal de Campina Grande, Av. Aprígio Veloso-882, Bodocongó, Campina Grande-PB 58429-900, Brazil

**Keywords:** scheelite tailings, ceramic formulations, mullite, physico-mechanical properties

## Abstract

New ceramic formulations based on scheelite tailing were developed, and their potential in the ceramic industry was evaluated. Green bodies with different contents of scheelite tailing (0–8 wt%) were sintered (1150 °C, 1200 °C, and 1250 °C) and characterized in terms of the main mineralogical phases, microstructure, and physico-mechanical properties. The mullite was the main phase identified in all sintered temperatures. This result was also ratified with the aid of scanning electron microscope (SEM) images, in which small needles of the mullite were detected. The presence of mullite is required because it contributes to increasing the mechanical resistance of the material. The physico-mechanical properties measured (water absorption, linear shrinkage, apparent porosity, and flexural strength) were compared to the ISO 13006, and the samples sintered at 1150 °C presented potential to be used as semi-stoneware, while those sintered at 1200 °C and 1250 °C can be employed stoneware and porcelain tiles, respectively.

## 1. Introduction

The scheelite is the main mineral source of the tungsten and may contain concentrations of the tungsten trioxide (WO_3_) between 0.08–1.5% [1,2]. Tungsten is an economically significant metal with numerous technological applications, such as high-temperature furnace components and lamp components, cutting and wear-resistant devices, alloys and specialty steels, electric and electronic applications, etc. [3,4,5]. As a consequence, scheelite’s mining activity is intense, profitable being that the principal mines of this mineral are located in China, Alaska, Mexico [5], Canada [6], Australia [7], Uzbekistan [8], and northeastern Brazil [9]. Despite the scientific, technological, and economic importance of mineral extraction, this activity generates a large number of tailings. When irregularly disposed of, the tailings can release harmful substances into the water, air, and soil, leading to a human health disorder. It is also worth noting that tailing management costs the mines companies since they are responsible for the transportation, treatment, and disposal.

In this way, several studies have addressed the incorporation of mines tailings in ceramic formulations. Among these studies, we can highlight the article published by Medeiros et al. [10], which studied the influence of quartzite tailing in partial substitution of feldspar in a ceramic formulation (clay, feldspar, quartz, and kaolin) to manufacture sanitary ware. In summary, the ceramic formulation containing up to 15% of the quartzite presented water absorption (~0.5%) and mechanical resistance values above 35 MPa, which are within the range recommended for sanitary ware. Scanning electronic microscopy images (SEM) also showed that the microstructure of the ceramic formulation developed is similar to the standard ceramic mass. Silva et al. [11] investigated the mineralogical, physical, and dielectric characteristics of the ceramic formulations contained industrial inorganic residues (kaolin and alumina), clay, and magnesium oxide. After sintering treatment (temperatures range between 950 °C and 1400 °C), the mullite and cordierite were the major mineralogical phases identified. The authors find that the ceramic formulations sintered at 1300 °C contained mullite with, their dielectric constant measured was close 5.7–6.4 at a frequency of 1 kHz. On the other hand, the ceramic formulation contained cordierite (sintered at 1350 °C) presented a low dielectric constant (0.8–1.2) and a loss tangent of 0.003–0.009 at 100 kHz. Finally, the authors reported that the temperature increased negatively and promoted a reduction of porosity, contributing directly to reducing the dielectric properties of the ceramic formulations investigated.

Although scheelite tailing has the potential to be used in civil construction, few studies have addressed the topic. The work published by Souza et al. [12] studied the mechanical behavior of mortars made with scheelite tailing in total replacement to the conventional aggregate in wet curing. In summary, the authors concluded that this tailing has favorable physical characteristics for use in tile mortars. Another work worth mentioning was published by Medeiros et al. [9], which aimed to characterize the scheelite tailing, identify its similarity with natural sand, and produce a tile mortar. In this work, besides the scheelite tailing, Portland cement type CPII-F32 and industrialized calcium hydroxide were used as a binder. After characterization by laser granulometry, apparent and relative density, X-ray fluorescence (EDX), X-ray diffraction (XRD), and thermal analysis, the authors concluded that the scheelite tailing had a chemical and physical characteristic similar to natural sand employed to manufacture tile mortars. Thermal analysis experiments, mechanical tests (tensile bond strength and compressive strength), SEM, and water absorption tests were accomplished and indicated that scheelite tailing could be used as fine aggregate to replace natural sand. Also, the ceramic formulations developed had the potential to be employed as a tile mortar.

In the manufacturing process of the ceramic pieces, the strict control of physical properties, such as porosity and water absorption, is crucial to its classification, characteristics, and potential applications. The ISO 13006 [13] is used to standardize ceramic formulation with ceramic tiles potential. Second, the ISO 13006, ceramic tiles can be classified into three groups: Group I (WA ≤ 3%). Group II (3% < WA ≤ 10%), and Group III (WA > 10%). Each group has subdivisions that take into account the method of manufacture (extruded and dry-pressed tiles). Regard dry-pressed tiles, the method used in this work, Group I is subdivided into Group Bl_a_ (WA ≤ 0.5%) and Group Bl_b_ (0.5% < WA ≤ 3%); and Group II in Group Bll_a_ (3% < WA ≤ 6%) and Bll_b_ (6% < WA ≤ 10%).

Despite the potential of scheelite tailings to be used in civil construction, few works address the topic. Therefore, the main objective of this work was to develop new ceramic formulations based on scheelite residues with the potential to be used in the manufacture of porcelain tiles, semi-stoneware, and stoneware. For this, all the raw materials used were characterized, compacted, and sintered at 1150 °C, 1200 °C, and 1250 °C. The sintered samples were then investigated to the main mineralogical phases, microstructure, and physico-mechanical properties (water absorption, apparent density, linear retraction, and flexural strength).

## 2. Materials and Methods

### 2.1. Raw Materials

The new ceramic formulations investigated in this work were prepared from the following raw materials: scheelite tailing (SR) derived from Mineração Tomaz Salustiano S.A., located in the of Currais Novos city, Rio Grande do Norte state (Brazil); kaolin (K) supplied by Rocha Minérios, located in Juazeirinho city, Paraíba state (Brazil); plastic clay (PC), quartz (Q) and feldspar (F) supplied by the industry Armil Mineração do Nordeste, located in Parelhas city, Rio Grande do Norte state (Brazil).

### 2.2. Samples Preparation and Sintering Treatments

The samples investigated in this research were prepared from 27 wt% kaolin, 29 wt% plastic clay, 11 wt% quartz, and different contents of scheelite tailing and feldspar. The nominal compositions and nomenclature of the investigated ceramic formulations are summarized in Table 1.

Before the sintering step, the raw materials were grounded at a ball mill (450 rpm and 60 min), sieved (0.180 mm), mixed, and wet-milled (7% moisture content by wt.). The mixture was then dried at 110 °C for 24 h and uniaxially pressed (Hydraulic press model CT-335, Servitech, Tubarão, Brazil) in a rectangular mold (60 mm × 40 mm × 7.0 mm). The pressing step was accomplished in two stages, in which the first stage, the mixture was pressed at 13.5 MPa for 10 s, and in the second stage, another pressing was carried out at 50 MPa for 20 s. The pressed samples were dried at 110 °C for 24 h and sintered in a conventional electric oven (FE 50 RP Controller, Flyever Equipment, São Carlos, Brazil). The sintering protocol consisted of heating the samples (30 °C·min^−1^) from room temperature to different temperatures (1150 °C, 1200 °C, and 1250 °C) followed by an isothermal treatment (40 min). After isothermal treatment, the electric oven was turned off, and the samples were cooled down according to the oven’s inertia.

### 2.3. Characterizations of Raw Materials

The chemical composition of raw materials was obtained by X-ray fluorescence (model EDX 720, Shimadzu, Kyoto, Japan). The mineralogical phases were identified by X-ray diffraction (XRD) using a diffractometer (model XRD 6000, Shimadzu, Kyoto, Japan) with Cu-Kα radiation in a 2 theta range from 5° to 60°, an angular step of 0.02, and a counting time of 0.5 s [14]. The experimental data were collected at room temperature. The diffraction peaks observed were indexed using the Search Match^®^ Program (Crystal Impact, Bonn, Germany) and the JCPDS database (ICDD, Newtown Square, United States).

The particle size distribution of the raw materials was determined using the laser diffraction granulometry method (model 1064 LD, CILAS, Orléans, France). The analysis was carried out in an aqueous medium in a proportion of 250 mL of distilled water for each 5 g of material. For the dispersion, a high-speed mechanical stirrer (17,000 rpm) was used for 10 min and sodium hexametaphosphate as a dispersing agent. Before the analysis, all raw materials were processed through a sieve (74 μm).

The thermal behavior was evaluated by thermogravimetric analysis (model TA 60H, Shimadzu, Kyoto, Japan) and differential thermal analysis (DTA) (model RB-3000, Instrumentec BP, Campinas, Brazil). In both, the heating rate used was 12.5 °C·min^−1^ and the air atmosphere.

### 2.4. Samples Characterizations after Sintering Treatment

The mineralogical phases formed after the sintering treatment were also identified with the aid of X-ray diffraction (XRD), following the same parameters previously described. The morphologies of crystals were analyzed using scanning electron microscopy (SEM) images (model SSX-550, Shimadzu, Kyoto, Japan).

The Archimedes method was used to measure water absorption (WA) and an apparent density (AD). Measurements of linear shrinkage (LS) and flexural strength at three points were also obtained. Each experiment was repeated 10 times for different samples. The procedures for carrying out these tests are described in other works [15,16,17].

## 3. Results and Discussion

### 3.1. Raw Materials Characterizations

**Figure 1:**
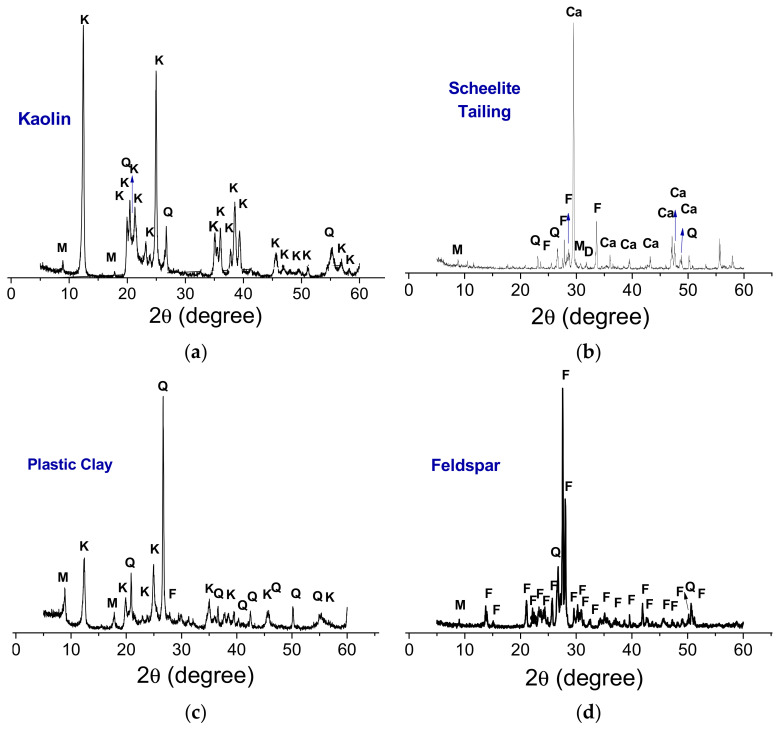
XRD patterns measured from raw materials (**a**) kaolin, (**b**) scheelite tailing, (**c**) plastic clay, and (**d**) feldspar. (M—Mica, K—Kaolinite, Q—Quartz, F—Feldspar, Ca—Calcite, D—Dolomite).

Figure 1 shows XRD patterns measured from raw materials (kaolin, plastic clay, quartz, feldspar, and scheelite tailing). Kaolinite (JCPDS 78-2110) was identified as the major crystalline phase in kaolin and plastic clay. On the other hand, mica (JCPDS 83-1808) and quartz (JCPDS 46-1045) were identified to a lesser extent. The plastic clay also showed a characteristic peak of potassium feldspar (JCPDS 84-0710). These peaks are similar to those reported by other authors [18,19]. The mineralogical phases detected in the feldspar were mica (JCPDS 83-1808), feldspar (JCPDS 84-0710), and quartz (JCPDS 46-1045). The scheelite tailing contains calcite (JCPDS 47-1743) as the predominant phase, and traces of quartz (JCPDS 46-1045), mica (JCPDS 83-1808), feldspar (JCPDS 84-0710), and dolomite (JCPDS: 89-5862).

Table 2 summarizes the chemical composition of raw materials. The main constituent oxides detected in kaolin and the plastic clay were SiO_2_ (45.7 wt% and 54.5 wt%, respectively) and Al_2_O_3_ (39.5 wt% and 27.5 wt%, respectively). Both are derived from the structure of clay minerals and free silica [20,21]. The high K_2_O content (4.0%) was detected in plastic clay. The high levels of CaO (44.7 wt%) and SiO_2_ (20.8 wt%) identified in the scheelite tailing (SR) indicate the presence of calcite and quartz in its composition. The amount of K_2_O (12.1 wt%) in feldspar indicates its potassium nature and contributes to forming a vitreous phase at low temperatures during the sintering step [20]. The high fire loss of scheelite tailing (15.8 wt%) is attributed to the thermal decomposition of calcium carbonate in calcium oxide and gas. According to Table 2, it can be observed that scheelite tailing will contribute to calcium and iron to the ceramic mass. However, the amount of iron will be lower than 1% in the formulation, which favors the achievement of fired bodies with a light color [22]; and the presence of a higher calcium content will contribute with a higher amount of liquid phase during sintering.

**Figure 2:**
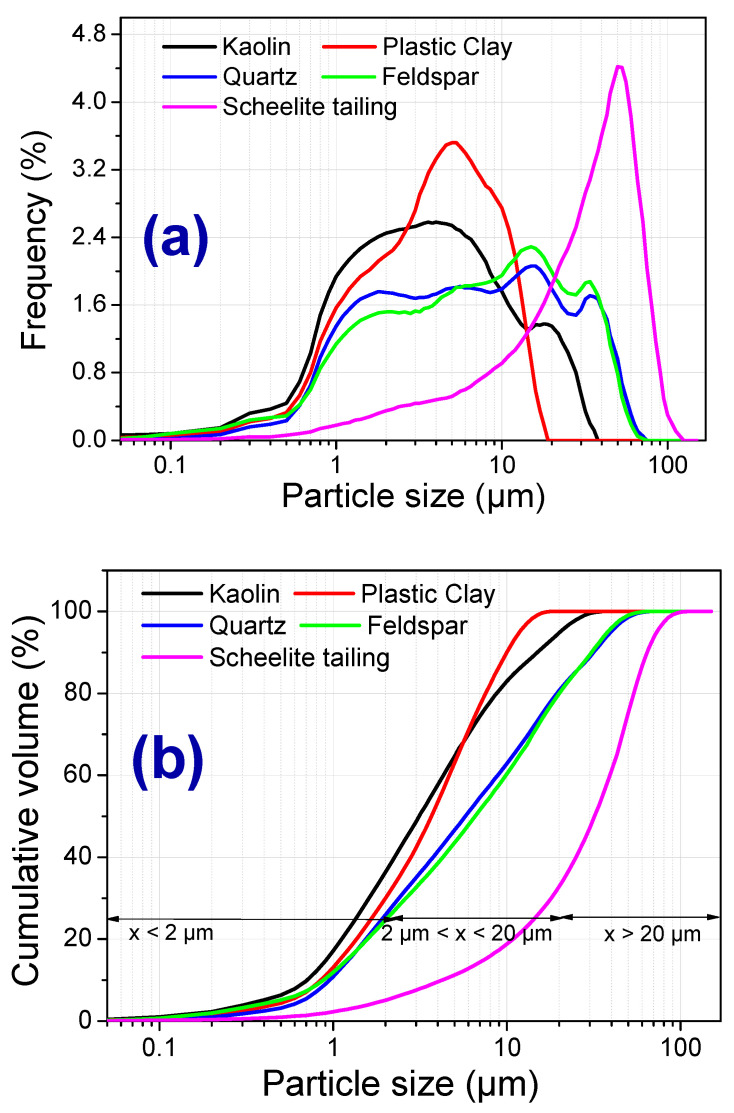
Granulometric distribution of raw materials. (**a**) simple frequency curves, and (**b**) accumulated frequency curves.

Figure 2 shows the particle size distribution measured from the raw materials and ceramic formulations before the sintering step. Among the raw materials, it is possible to see that the scheelite tailing had the largest average particle diameter (33.6 μm) and a fraction of particles with diameters above 20 μm (67.0% of the accumulated volume). For the other raw materials, the most considerable fraction of particles measured was between 2 and 20 μm, with 58.1%, 70.0%, 55.5%, and 54.9% of accumulated volume for kaolin, plastic clay, quartz, and feldspar, respectively. It is known that the particle size distribution significantly influences the packing of particles and the product sintered. The smaller particle size leads to greater surface area and reactivity between the particles, which favors the reactions kinetics and the diffusion process during the phase transformations [17,23,24]. This contributed to limit the number of tailings added to ceramic mass due to the coarser particle size of the waste compared with feldspar.

**Figure 3:**
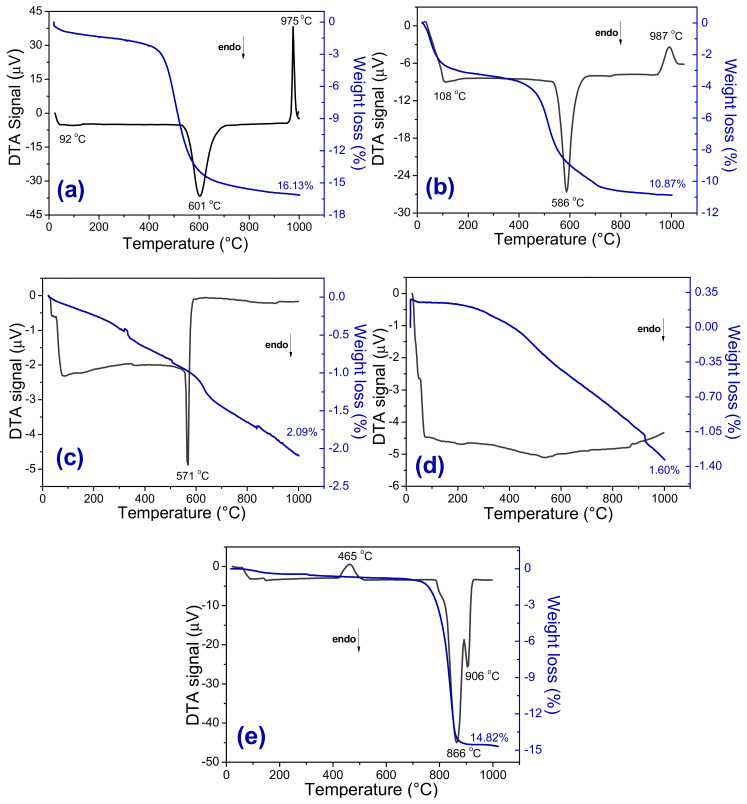
DTA and TG curves measured from the raw materials. (**a**) kaolin, (**b**) plastic clay, (**c**) quartz, (**d**) feldspar, and (**e**) scheelite tailing. All samples were heated with a heating rate of 12.5 °C·min^−1^.

Figure 3a–e shows differential thermal analysis (DTA) and thermogravimetric (TG) curves measured from raw materials. In the DTA curves measured from the kaolin (Figure 3a) and plastic clay (Figure 3b), it was possible to see two endothermic events and one exothermic. The endothermic events are related to free water loss (~92 °C for kaolin and ~108 °C for plastic clay) and loss of hydroxyls (~601 °C for kaolin and ~586 °C for plastic clay) of the octahedral sheet present in the clay structure. The exothermic peak reached the maximum at ~975 °C and 987 °C for kaolin and plastic clay, respectively, and refers to the mullite nucleation [25]. In the quartz DTA curve (Figure 3c), the endothermic peak observed occurred at 571 °C and is associated with alpha-quartz transformation into beta-quartz [26]. No transition was observed in the DTA curve measured from feldspar (Figure 3d). The total losses of mass for kaolin, plastic clay, quartz, and feldspar were 16.1%, 10.9%, 2.1%, and 1.6%, respectively, which corresponds to the water’s losses hydroxyls of the clay minerals. The DTA curve measured from the scheelite tailing (Figure 3e) shows an exothermic peak at ~465 °C, which may be related to the crystallization of amorphous calcium carbonate or with the polymorphic transformation of vaterite to calcite [27]. In Figure 3, two endothermic peaks were observed (866 °C and 906 °C) and are related to the carbonate decomposition. The total mass loss observed for the scheelite tailing was 14.8%. The more significant loss of mass occurred between 700 °C and 850 °C, which is related to the release of CO_2_ due to the decomposition of the calcite and dolomite minerals.

**Figure 4:**
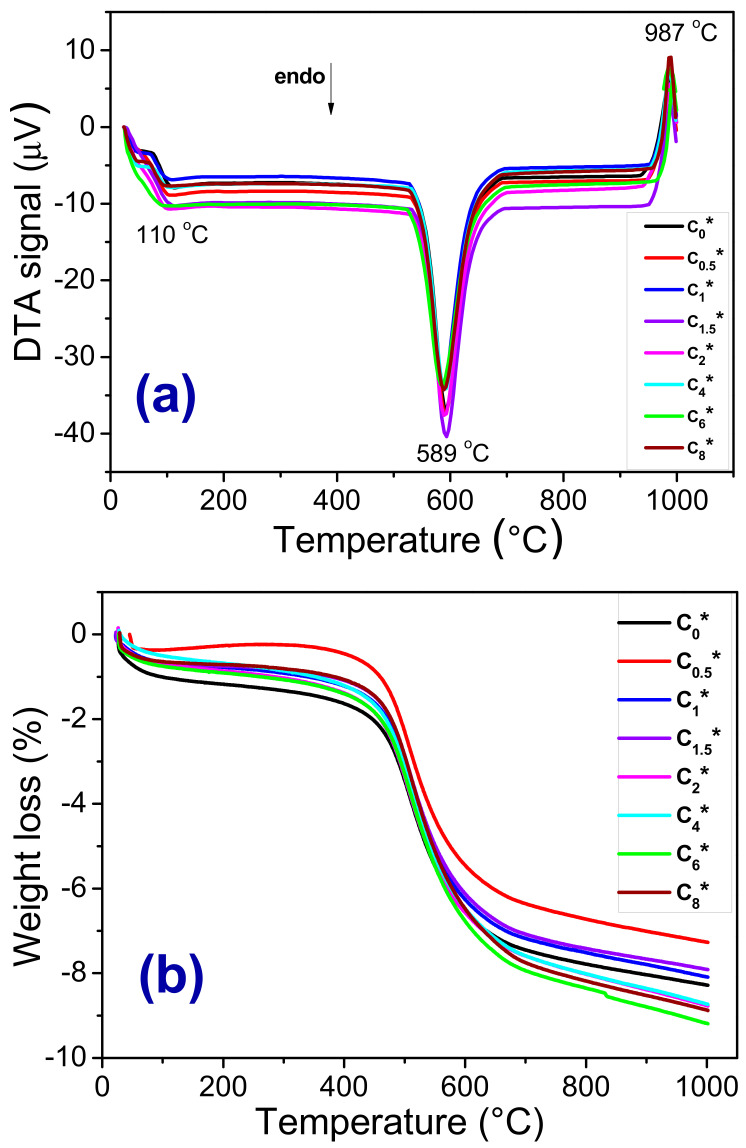
DTA (**a**) and TG (**b**) curves measured from the ceramic formulations before sintering (C_0_* C_0.5_*, C_1_*, C_1.5_*, C_2_*, C_4_*, C_6_*, and C_8_*). All samples were heated with a heating rate of 12.5 °C·min^−1^.

Figure 4a–b show DTA and TG curves of the ceramic formulations before sintering (C_0_*, C_0.5_*, C_1_*, C_1.5_*, C_2_*, C_4_*, C_6_*, and C_8_*). In general, all DTA and TG curves follow the same behavior, which indicates that the incorporation of scheelite tailing, up to 8 wt%, did not significantly affect the thermal behavior of the material and did not influence the mullite nucleation temperature. In the DTA curves (Figure 4a), two endothermic peaks and one exothermic peak were identified. The first endothermic peak occurred at 110 °C and is related to the free and adsorbed water loss, and the second (~589 °C) is associated with the loss of hydroxyls from kaolin and plastic clay, as well as the transformation of α-quartz into β-quartz. At around 987 °C, the exothermic peak is related to the mullite nucleation [28]. All ceramic formulations showed a total loss of mass (Figure 4b) between 7.9% and 9.0%.

### 3.2. Crystalline Phases, Microstructural and Physicomechanical Properties

**Figure 5:**
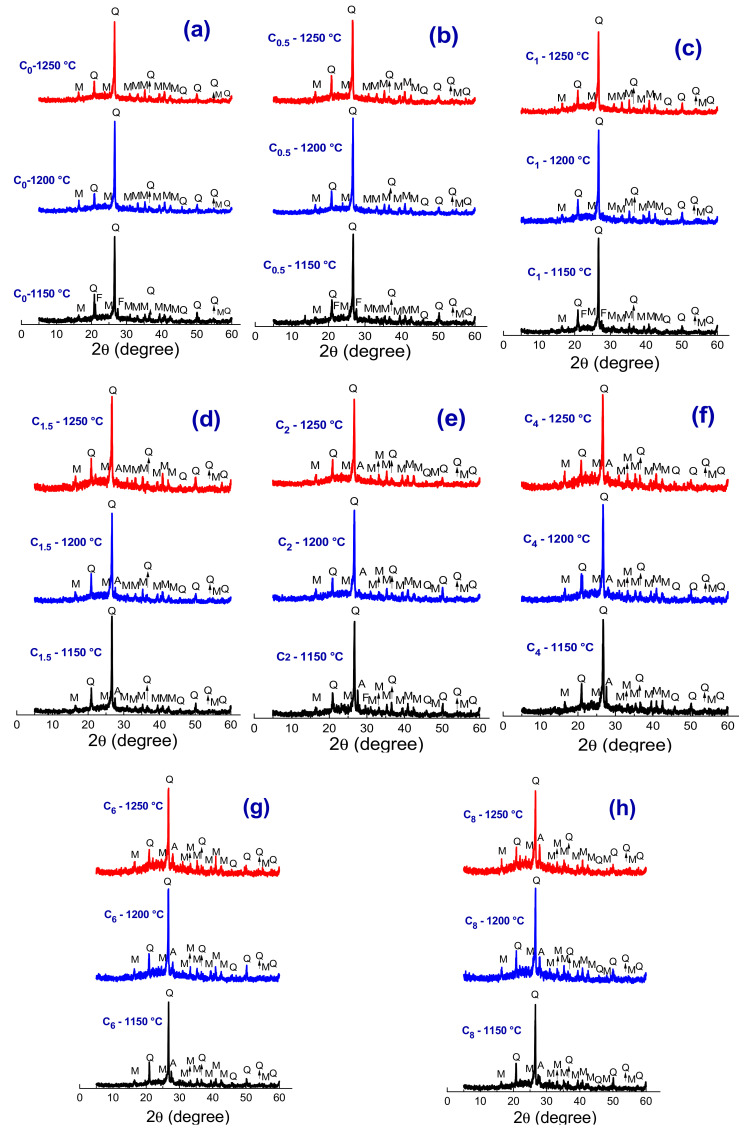
XRD patterns of ceramic formulations sintered at 1150 °C, 1200 °C, and 1250 °C. (**a**) C_0_, (**b**) C_0.5_, (**c**) C_1_, (**d**) C_1.5_, (**e**) C_2_, (**f**) C_4_, (**g**) C_6_, and (**h**) C_8_. (M—Mullite, Q—Quartz, A—Anorthite).

Figure 5a–f shows XRD patterns of sintered samples (C_0_, C_0.5_, C_1_, C_1.5_, C_2_, C_4_, C_6_, and C_8_). For all investigated samples, it was possible to identify mullite (JCPDS 79-1276), quartz (JCPDS 46-1045), and anorthite (JCPDS 09-0465). The presence of a small amorphous halo was also noticed between 15 ° and 25 ° (2θ), which is related to the glass phase. This glassy phase is probably formed due to excess SiO_2_ in the ceramic formulations [29]. The amorphous halo area reduced with the increasing sintering temperature, which may be directly related to the formation of mullite and, consequently, a decrease in the glass phase.

In addition, in Figure 5, it was possible to observe that in the diffractogram measured from the C_0_, C_1.5_, and C_2_ samples (Figure 5a–c) treated at 1150 °C, the presence of feldspar (JCPDS 84-0710). Here, it is worth mentioning that the C_0.5_ and C_1_ samples also showed feldspar peaks since their XRD patterns are similar to those measured from the C_0_ sample. The presence of remaining feldspar indicates that this sintering temperature (1150 °C) was not enough to promote its complete melting since the mentioned ceramic formulations have a higher content of this material (see Table 1). Still talking on the C_0_, C_0.5_, C_1_, C_1.5_, and C_2_ samples, the disappearance of feldspar with increasing sintering temperature was observed. The mullite and quartz phases are related to the clay mineral presented in clays and quartz low dissolution kinetics [30]. Mullite is considered one of the most important phases in the production of ceramic materials, due to its exceptional properties, such as high melting point (~1830 °C), high resistance to thermal shock, chemical attack, and low coefficient thermal expansion (~4.5 × 10^−6^ K^−1^) [31]. The increase in temperature to 1250 °C led to a decrease in the quartz peaks’ intensity, indicating its partial dissolution.

The ceramic formulations with higher scheelite tailing contents, i.e., above 4 wt% (Figure 5d–f), presented in addition to the mullite and quartz phases a low-intensity peak associated with the anorthite phase (A). The higher content of scheelite tailing in these ceramic formulations may have favored the appearance of anorthite since this residue has a chemical composition rich in CaO, as evidenced by the result of the chemical analysis (see Table 2). Anorthite (CaO·Al_2_O_3_·2SiO_2_) is the lime-rich end member of the plagioclase feldspar series and has great potential as a reinforcing phase ceramic tiles and porcelains [32,33]. It has a coefficient of thermal expansion higher than mullite, on the order of 10^−15^–10^−6^ °C^−1^, what may cause a prestress state on the body due to the difference in the coefficient of thermal expansion of this phase and the vitreous phase, with a compression component enabling an increase of the body strength [34].

**Figure 6:**
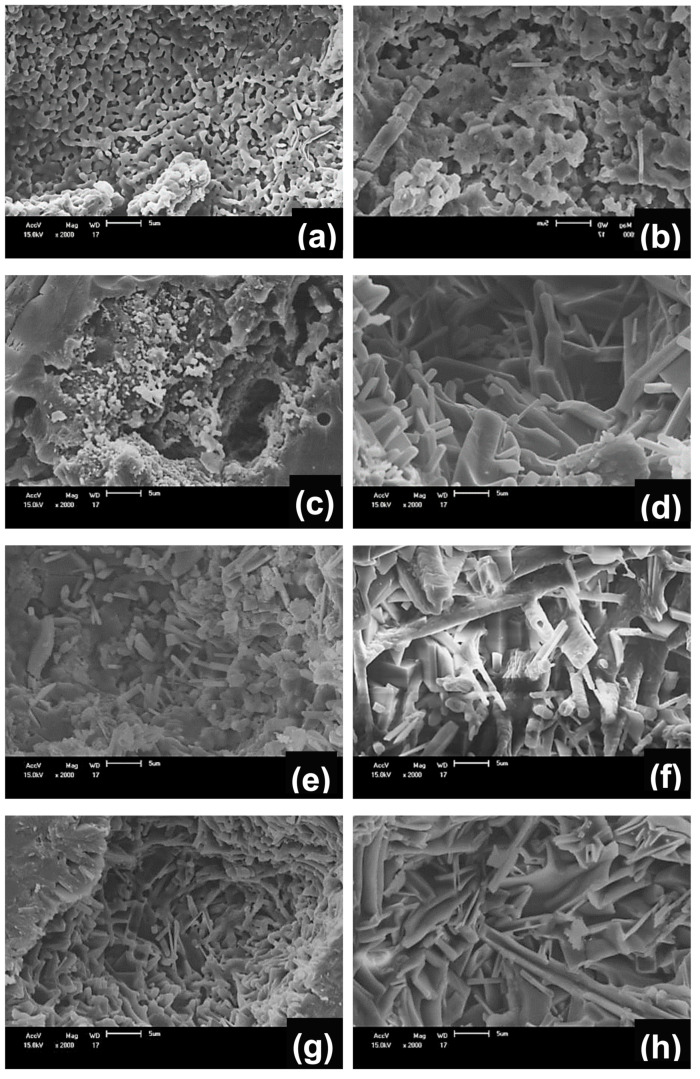
SEM micrographs of samples treated at 1150 °C (**a**) C_0_, (**b**) C_0.5_, (**c**) C_1_, (**d**) C_1.5_, (**e**) C_2_, (**f**) C_4_, (**g**) C_6_, and (**h**) C_8_.

Figure 6a–h shows the SEM images acquired from C_0_, C_0.5_, C_1_, C_1.5_, C_2_, C_4_, C_6_, and C_8_ samples sintered at 1150 °C. In general, these samples showed irregular microstructures. The microstructure on the C_0_ sample (namely, without scheelite tailing) sintered at 1150 °C (Figure 6a) is formed by small needles of mullite, possibly primary mullite, as this is the characteristic phase of this temperature range [35]. Generally, mullite forms a network of structures in needles that increase the material’s resistance [36].

**Figure 7:**
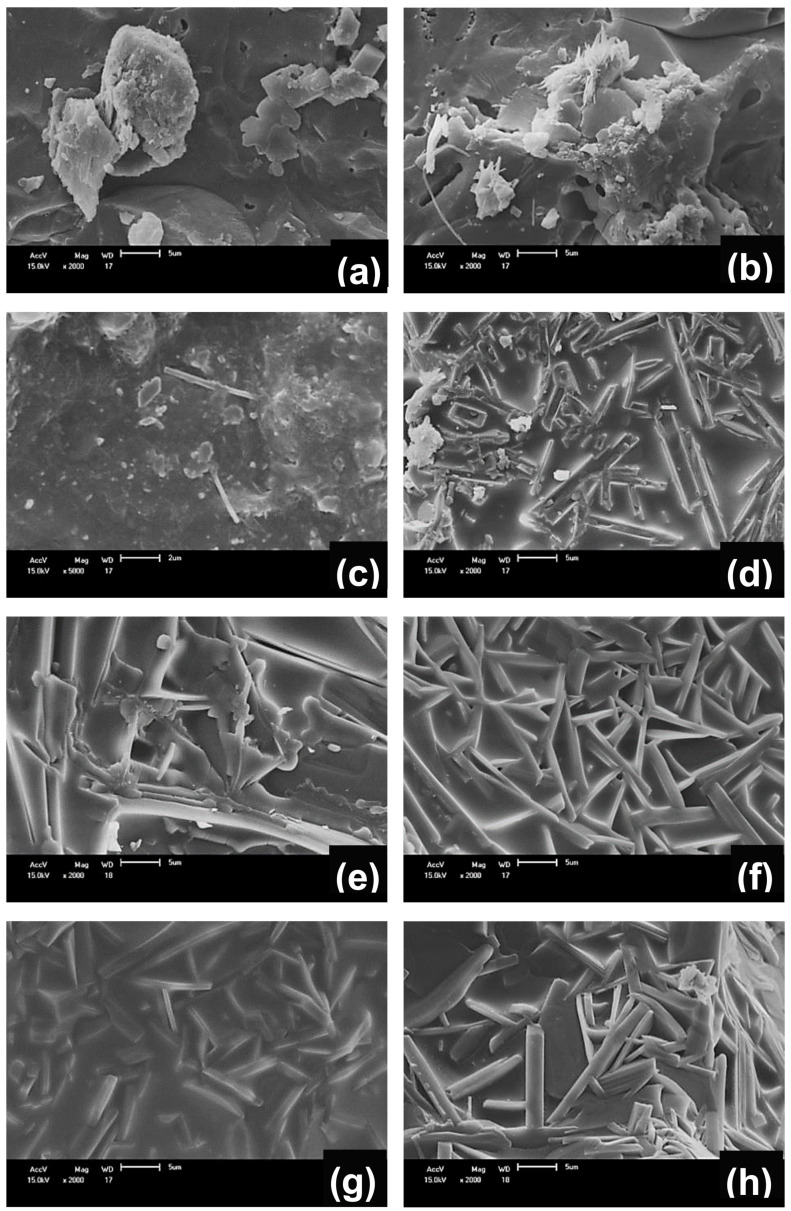
SEM micrographs of samples treated at 1250 °C (**a**) C_0_, (**b**) C_0.5_, (**c**) C_1_, (**d**) C_1.5_, (**e**) C_2_, (**f**) C_4_, (**g**) C_6_, and (**h**) C_8_.

The incorporation of scheelite tailing in the ceramic formulation favored the growth of mullite crystals (Figure 6b–h and Figure 7b–h). This behavior occurs because generally, the presence of impurities (such as CaO present in the scheelite tailing) acts to reduce the temperature of formation of the metastable siliceous liquid, favoring mullitilization. The mullite crystal growth is probably attenuated in the C_0_ sample due to a less viscous liquid phase, which does not favor the dissolution of silica and alumina [37]. Anorthite crystals were not observed because they have isometric round morphology with a size smaller than those of mullite [29] and probably are immersed in the vitreous phase.

**Figure 8:**
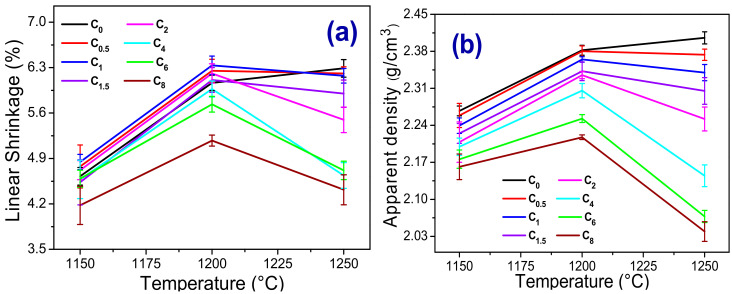

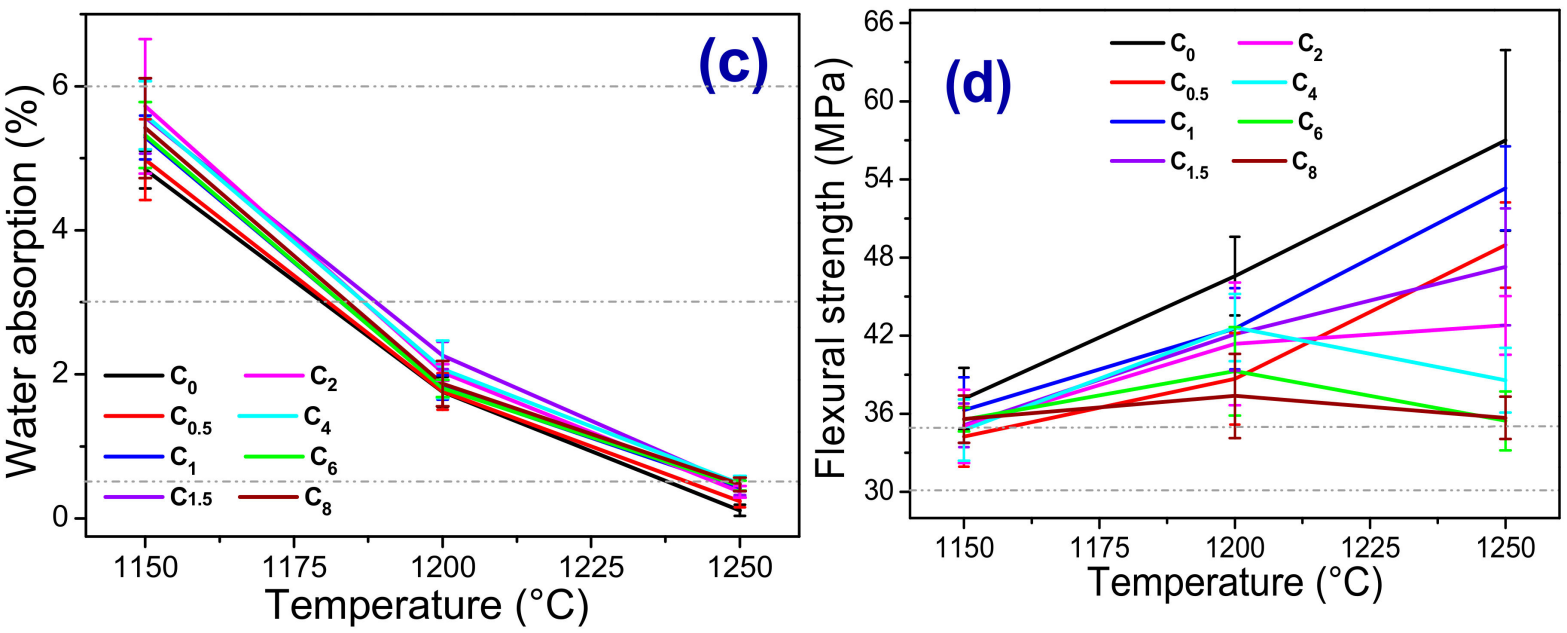
Physical-mechanical properties depending on the temperatures measured from the samples after the sintering treatment. (**a**) Linear shrinkage, (**b**) Apparent density, (**c**) Water absorption, and (**d**) Bending stress.

Figure 8a–d shows the results of the physical-mechanical properties of sintered samples. For all ceramic formulations studied, it was observed that the linear shrinkage (LS) (Figure 8a) increases with increasing sintering temperature from 1150 °C to 1200 °C and decreases when this temperature increases from 1200 °C to 1250 °C. The increase in LS (1150 °C → 1200 °C) occurs due to the higher production of liquid phase with the increase in temperature, which favors densification (Figure 8b) and consequently leads to more significant retraction of the samples and reduction of porosity [38]. However, when the temperature increases from 1200 °C to 1250 °C, LS tends to decrease due to the presence of a high amount of low viscosity liquid phase and the expansion of the gas in the closed porosity of the material. The apparent density (AD) results (Figure 8b) follow the same temperature-dependence shown for linear shrinkage, which is already expected since these properties’ values are directly proportional. The decrease in shrinkage and density was more pronounced for those samples with a residue content above 2 wt%. This behavior can be related to the presence of calcium scheelite tailing, which decreases the mass’s eutectic temperature, improving the amount of liquid phase and probably decreasing its viscosity (see Figure 1).

During the sintering process, the calcite decomposes into CaO and CO_2_. The CO_2_ increases the pressure inside the material’s closed pores compromises the densification and consequent retraction of the material. The decrease in water absorption (WA) (Figure 8c) with the increase in sintering temperature is related to the filling of the pores by the liquid phase that was formed in greater quantities at higher temperatures [17,24]. This implied a decrease in the apparent porosity and a consequent increase in mechanical strength. In general, for all investigated ceramic formulations, the increase in temperature by up to 1200 °C led to an increase in flexural strength (FS) (Figure 8d). From 1200 °C to 1250 °C, the mechanical resistance increases for compositions with residue content up to 2 wt% (C_0.5_, C_1_, C_1.5_, and C_2_ samples) and decreases in the other compositions (C_4_, C_6_, and C_8_ samples), corroborating with the results of linear shrinkage and apparent density. The highest flexural strength measured for waste containing samples was achieved in C_1_, 53 MPa sintered at 1250 °C. According to the criteria established by ISO 13006 [39] all compositions sintered at 1150 °C can be classified as semi-stoneware (3.0% < WA < 6.0% and FS ≥ 22 MPa), those sintered at 1200 °C as stoneware (0.5% < WA < 3.0% and FS ≥ 30 MPa) and finally, those sintered at 1250 °C as porcelain tiles (WA ≤ 0.5% and FS ≥ 35 MPa).

## 4. Conclusions

The incorporation of scheelite tailing in ceramic formulations was investigated to produce porcelain, stoneware, and semi-stoneware. SEM images obtained from the sintered samples (C_0_, C_0.5_, C_1_, C_1.5_, C_2_, C_4_, C_6_, and C_8_) revealed mullite crystals in which they contribute to the increase in mechanical strength.

The sintering temperature significantly influenced the physical-mechanical properties (LS, AD, WA, and FS). The increase in temperature from 1150 °C to 1200 °C resulted in a decrease in the experimental values of water absorption and increased values of linear shrinkage, apparent density, and flexural strength. However, when the temperature increased from 1200 °C to 1250 °C, the mechanical resistance increased for compositions with up to 2 wt% of residue and decreased for those with content above 4 wt%. In general, all compositions showed flexural strength greater than 30 MPa. According to the criteria established by the international standard ISO 13006, the parts sintered at 1250 °C can be used as porcelain tiles; those sintered at 1200 °C as stoneware and those sintered at 1150 °C as semi-stoneware.

## Figures and Tables

**Table 1:** Nomenclature and nominal compositions (wt%) of the ceramic formulations investigated in this work.

Compositions	Raw Materials				
	Kaolin	Plastic Clay	Quartz	Feldspar	Scheelite Tailings
C_0_	27	29	11	33.0	-
C_0.5_	27	29	11	32.5	0.5
C_1_	27	29	11	32.0	1.0
C_1.5_	27	29	11	31.5	1.5
C_2_	27	29	11	31.0	2.0
C_4_	27	29	11	29.0	4.0
C_6_	27	29	11	27.0	6.0
C_8_	27	29	11	25.0	8.0

**Table 2:** Chemical compositions of raw materials (kaolin, plastic clay, quartz, feldspar, and scheelite tailing).

Raw Materials	Oxides (%)								
	SiO_2_	Al_2_O_3_	Fe_2_O_3_	K_2_O	MgO	CaO	Na_2_O	Others	LF ^1^
Kaolin	45.7	39.4	0.5	0.9	-	-	-	0.2	13.3
Plastic clay	54.5	27.4	2.6	4.0	1.5	0.8	-	1.4	7.8
Quartz	95.0	2.4	0.2	0.1	-	-	-	0.3	2.0
Feldspar	62.0	19.5	-	12.1	-	-	2.4	2.4	1.6
Scheelite tailing	20.7	7.0	6.8	0.4	2.6	44.6	-	2.1	15.8

^1^ LF: Loss to fire determined by burning at 1000 °C, after drying at 110 °C.
